# Whole Blood Based Multiparameter Assessment of Thrombus Formation in Standard Microfluidic Devices to Proxy In Vivo Haemostasis and Thrombosis

**DOI:** 10.3390/mi10110787

**Published:** 2019-11-16

**Authors:** Isabella Provenzale, Sanne L. N. Brouns, Paola E. J. van der Meijden, Frauke Swieringa, Johan W. M. Heemskerk

**Affiliations:** Department of Biochemistry, Cardiovascular Research Institute Maastricht (CARIM), Maastricht University, 6229 ER Maastricht, The Netherlands

**Keywords:** collagen, platelet, thrombus

## Abstract

Microfluidic assays are versatile tests which, using only small amounts of blood, enable high throughput analyses of platelet function in several minutes. In combination with fluorescence microscopy, these flow tests allow real-time visualisation of platelet activation with the possibility of examining combinatorial effects of wall shear rate, coagulation and modulation by endothelial cells. In particular, the ability to use blood and blood cells from healthy subjects or patients makes this technology promising, both for research and (pre)clinical diagnostic purposes. In the present review, we describe how microfluidic devices are used to assess the roles of platelets in thrombosis and haemostasis. We place emphasis on technical aspects and on experimental designs that make the concept of “blood-vessel-component-on-a-chip” an attractive, rapidly developing technology for the study of the complex biological processes of blood coagulability in the presence of flow.

## 1. Introduction

Starting from the mid 70-tees, the laboratories of Baumgartner, Badimon and Sixma pioneered in the development of flow chamber devices to assess platelet adhesion and activation at surfaces composed of extracellular matrix components [1,2,3,4]. Already in early studies where whole-blood was flowed through a parallel-plate flow chamber at defined shear conditions, it became clear that the build-up of a thrombus observed in these devices resembled the thrombi formed in a damaged blood vessel [5]. It further appeared that this thrombus formation was sensitive to the presence in the blood of anticoagulant or antiplatelet agents, indicating that this method also senses haemostatic (stopped bleeding) activity of the blood sample. Indeed, in a recent synthesis approach of 1514 published studies of arterial thrombus formation in mice, it was established that thrombus formation on a collagen surface in flow chambers correlates with the outcome of experimental arterial thrombosis and of tail bleeding times [6]. Accordingly, such ‘flowed-blood-on-a-chip’ chambers can be considered as a highly informative technology in the broad field of thrombosis and haemostasis.

In particular parallel-plate microfluidic chambers, using small amounts of blood, have emerged as devices suited to study a wide range of blood-related processes. In combination with multicolour fluorescence microscopy, they allow the real-time visualisation of adhered platelets, fibrin and vascular cells under defined flow and shear conditions. Especially the ability to use primary human cells and patient blood samples makes whole blood microfluidics a promising tool for innovative blood research and future clinical applications. After a general description of the main (patho)physiological interactions of platelets and coagulation factors with a vessel wall, we describe how microfluidic devices can be used to unravel and assess those interactions.

## 2. Vessel Wall-Blood Component Interactions in Haemostasis and Thrombosis

The physiological process of haemostasis (vascular plug formation to stop bleeding) and pathophysiological thrombosis (intravascular formation of an occlusive thrombus resulting in heart or brain infarctions) are tightly balanced processes. Haemostasis and arterial thrombosis have several regulatory aspects in common, as both result from the crosstalk between vessel wall, platelets and the coagulation system. Thus, genetic mouse models have shown that functional defects in either platelet or coagulation activation or in endothelial-related vascular changes can all lead to impaired experimental thrombosis, frequently accompanied by abnormal haemostasis [6].

Below we briefly describe key interactions of platelets and coagulant factors with relevant vascular components. For a more extensive discussion on this topic, we will refer to earlier in-depth reviews [7,8]. Under arterial flow conditions, platelets adhere to an exposed subendothelial matrix, enriched in collagen and von Willebrand factor (VWF), before they become activated. Activation and subsequent aggregation of the platelets leads to the formation of a plug or thrombus, as well as in the release of a variety of autocrine substances enforcing this process. A dysfunction in each of these steps may result in impaired thrombosis and/or bleeding phenotype.

In a damaged vessel wall, also coagulation-promoting substances become exposed to the blood stream. These include tissue factor (TF), stimulating the extrinsic coagulation route, and collagen, which stimulates the intrinsic coagulation route [7,9]. In the subsequent reactions, the coagulation activation interacts with platelet activation processes. Thrombin that is generated via the TF pathway acts as a potent activator of platelets via the protease activated receptors (PAR). Conversely, highly activated platelets expose the negatively charged phospholipid, phosphatidylserine, which provides a membrane surface that strongly accelerates the coagulation cascade by stimulating factor Xa and thrombin generation [10,11]. Thrombin furthermore cleaves fibrinogen into fibrin, which consolidates the platelet aggregates to a stable platelet-fibrin clot [12,13]. Clot lysis or fibrinolysis is mediated by tissue-type plasminogen activator (tPA), which induces activity of the fibrinolytic protease plasmin [7].

In an intact vessel wall, the endothelium acts to potently suppress platelet as well as coagulation reactions [7]. Strongly platelet inhibitory molecules released by endothelial cells are prostacyclin [14,15] and nitric oxide [16]. In addition, the endothelial surface contains membrane-bound ecto-nucleotidases (CD39/CD73), which function to degrade platelet-activating ATP and ADP [17]. Anticoagulant properties of the endothelium are provided by the thrombin-inactivating thrombomodulin and by tissue factor pathway inhibitor (TFPI) [7,18]. In recent years, it has become clear that under conditions of vascular inflammation an unbalanced crosstalk is present between the ‘inflamed’ endothelium allowing limited activation of platelets and the coagulation system [19].

The blood fluid dynamics tightly regulate platelet adhesion to a damaged or inflamed vessel wall. The local wall-shear rate, defined as the force per unit area generated by flowed blood at a surface (Box A1), appears to tightly regulate platelet deposition [20], and also influence the kinetics of blood coagulation and fibrin clotting [21]. Herein, VWF—produced by endothelial cells—acts as an intermediating bioactive protein that controls the extent of platelet adhesion at high wall-shear rates [22]. Accordingly, at rates of 1000 s^−1^ and above, platelet deposition becomes markedly enhanced by adhesion to immobilised VWF. This results in stable adhesion depending on the combined receptors, GPIb-IX-V and integrin α_IIb_β_3_ [7,20,23]. This shear-dependent platelet adhesion can trigger the release of autocrine mediators, which mediate platelet aggregation and ultimately platelet procoagulant activity, stimulating the generation of fibrin [11,20,24].

Aberrant blood flow conditions affect platelet as well as coagulation processes, may lead to a dysfunctional endothelium, and can be associated with thrombo-haemorrhagic complications. Already two decades ago, shear-dependent platelet aggregation could be linked to thrombotic disorders, among which acute myocardial infarction, unstable angina and stroke [20,25,26]. In addition, endothelial cells that were programmed to an atherogenic phenotype—linked to inflammatory and oxidative responses and reduced release of nitric oxide and prostacyclin—appeared to be more thrombogenic as well [27]. Whereas in in vitro endothelial cells that are exposed to physiological flow and shear conditions act as a thromboprotective surface, this was no longer the case for endothelial cells subjected to stenotic, pathophysiological shear rates [28].

## 3. Whole Blood Microfluidics to Investigate and Measure Platelet Activation

During the last two decades, custom-made and commercial flow devices have widely been used to study platelet activation processes in flowed whole blood [4]. In the so-called parallel-plate flow chambers, platelets adhere to an immobilised vascular component —often collagen—, and then form aggregates or thrombi. Several useful devices have been developed, consisting of a small-sized high-precision, rectangular flow channel, operating with a push or pull pump system to perfuse whole blood through the chamber at a well-defined flow and shear rate (Table A1). When anticoagulated blood is used, preferentially at physiologically high (millimolar) CaCl_2_ and MgCl_2_ concentrations, the thrombi are formed as a result of platelet activation only, as the generation of thrombin and fibrin is prevented. The role of flowing red blood cells is to margin platelets to the parallel-plate sides of the flow channel whereas, at least in non-pathological blood samples, leukocytes do not participate in flow-dependent platelet adhesion and aggregation. Using these flow devices, the thrombogenic activity or capacity can easily be determined of human and animal blood. Practical possibilities and limitations of the technology are more extensively discussed elsewhere [29].

By applying adapted surface coating procedures, the platelet-activating effects can be established of different vascular proteins, such as collagen type-I and laminin. By simultaneously coating multiple surfaces as an array of small “microspots” (1 mm in diameter) [30], it is possible to assess platelet responses to several of vascular proteins in a single flow run (Box A2). Using the approach of microspot-based high-throughput measurements, we and others have been able to collect a considerable amount of information on the platelet quantitative and qualitative traits in blood from patients with a given bleeding diagnosis [30,31] as well as from fully genotyped healthy subjects [32,33]. In addition, we could establish the altered platelet properties in knockout mice in a thrombo-inflammatory setting [34].

**Figure 1:**
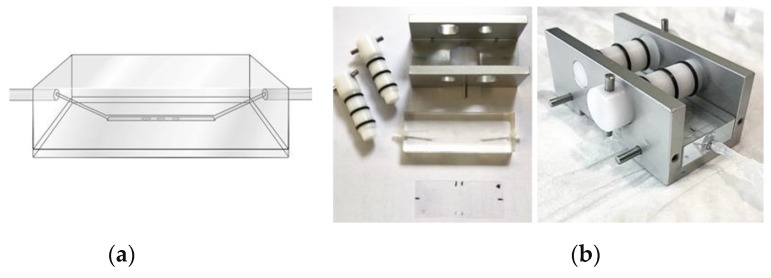
Design and assembly of the Maastricht flow chamber. (**a**) Schematic representation of the parallel plate chamber consisting of a channel, engraved into a transparent polycarbonate block. To prevent flow disturbances, the inlet and outlet ports reach the chamber via 20° angles. (**b**) The flow chamber block mounted onto a coated glass coverslip, and assembled in an aluminium holder. Two self-tapping clamping bolts are used to fix the coverslip to the polycarbonate block, thus preventing leakage. Original drawing and photographs.

Light-transparent flow chambers are required to establish precise control of the blood flow in thrombus formation, usually in combination with brightfield and/or fluorescence microscopy (Box A1). The polycarbonate Maastricht chamber, used in our laboratory [29], consists of a precision etched insert engraved in a transparent polycarbonate block (Figure 1a). A tubular inlet and outlet are connected to the actual chamber at low 20° angles, which prevent flow disturbations around the flat, parallel-plate measurement area. An advantage of such small size chambers is that they can operate with small blood volumes (0.5 mL), flowed in single pass.

For precision microspot-based assays, stringent control of the thrombogenic coating material is required. From this perspective, injection of a collagen solution into the chamber, and subsequent check on how much adheres to the surface is not the best option. An advantage of the Maastricht chamber is that it is covered by a disposable rectangular glass coverslip, which can be precisely coated with small microspots (1 mm in diameter) of collagen and/or other matrix materials. After blocking of the uncovered glass surface, the microspots will act as the only biologically active part of the chamber. An aluminium holder is used, containing two self-tapping clamping bolts, to fix and tighten the glass coverslip onto the chamber in a leak free manner (Figure 1b). This device is meanwhile used by multiple laboratories to characterise for instance the platelets from selected patients or from genetically modified mice with a prothrombotic or bleeding propensity [34,35,36,37,38]. A comparative analysis of 38 mouse strains, in which specific platelet genes had been knocked-out and blood samples were compared using the same device, has pointed to a key role of multiple proteins of the platelet GPVI signalling cascade in the process of thrombus formation [39]. Blood flow at high (arterial) or low (venous) wall shear rates can be achieved with a non-pulsating syringe pump (Box A1).

Commercial parallel-plate flow chambers and microcapillaries are also in use (e.g., Ibidi, Venaflux, Bioflux, Glycotech), although these often lack the ability of controlled application of the thrombogenic surface [40,41]. For a list of specific advantages and disadvantages of such commonly used devices, we like to refer to a previous overview [42]. In the last decade, several groups have experimented with microfluidic chambers made of soft polymeric materials, in particular polydimethyl siloxane (PDMS). Such PDMS chambers have as an advantage that complex shaped flow channels can be designed in a user-defined way [21,28]. By employing straight and rectangular PDMS chambers that were carefully inspected for irregularities (removing all visible obstructions from the flow channels!), comparable responses of platelet deposition and aggregation were measured as with the Maastricht chamber [43]. Relevant procedures on blood handling, flow chamber use, microscopic fluorescence imaging, and on image analysis are depicted in Box A2.

**Figure 2:**
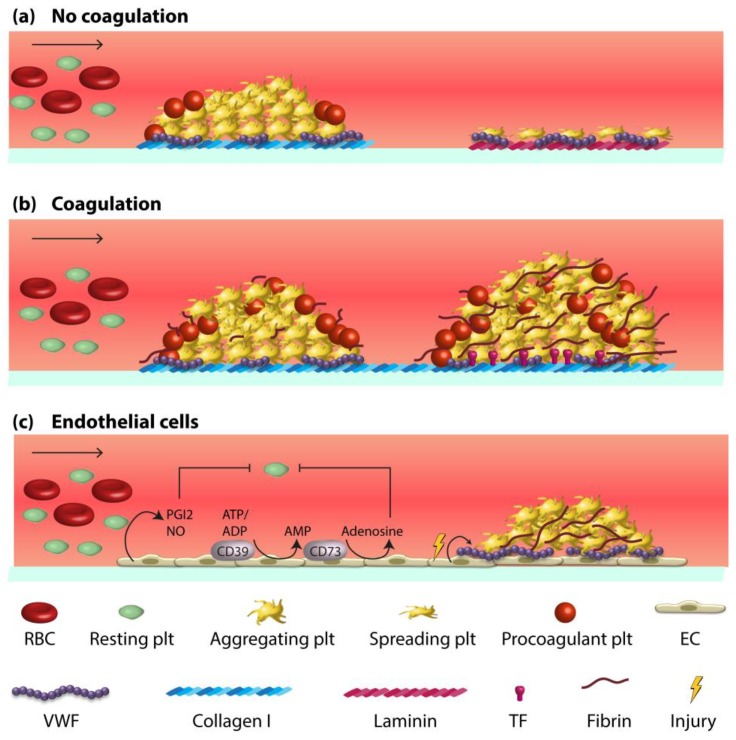
Schematic mechanisms of thrombus build-up obtained from flow-based assays. (**a**) Different types of thrombi formed under flow on microspotted platelet-adhesive surfaces. Coated fibrillar collagen (left) generates larger aggregates with procoagulant platelets, whereas laminin (right) induces deposition of a monolayer of spread platelets without procoagulant activity. Plasmatic VWF(purple) binding to the matrix proteins mediates the slowing down of platelets via the receptor GPIb-IX-V. (**b**) Under coagulant conditions, platelet aggregates on collagen only (left) form contracted thrombi containing fibrin due to activation of the intrinsic coagulation pathway. On collagen/TF (right), the extrinsic coagulation pathway is also triggered, causing contracted thrombi with more massive production of a fibrin network. In either case, procoagulant platelets (with surface exposure of phosphatidylserine) accelerates the thrombin and fibrin formation. (**c**) Endothelial cells (left) prevent platelet activation by releasing prostacyclin (PGI_2_) and nitric oxide (NO). In addition, membrane-bound ecto-nucleotidases (CD39/CD73) exert anti-platelet functions by degrading ATP/ADP to adenosine. An inflamed endothelium (right) shows enhanced release of strings of ultra-large VWF multimers, which can anchor to the endothelial surface and initiate platelet adhesion via GPIb-IX-V. The strings are cleaved by the plasmatic protease ADAMTS-13. Original drawing.

The nature of the (vascular) protein used for coating is an important determinant for the type of thrombus formed by flowed platelets {De Witt, 2014 #240]. Noteworthy, only fibrillar collagens cause the formation of multi-layered platelet thrombi, while laminins rather produce a monolayer of spread platelets (Figure 2a).

## 4. Whole Blood Microfluidics to Study Platelet and Coagulation Activation

Mouse models of in vivo arterial and venous thrombosis point to a quasi-simultaneous accumulation of platelets and fibrin at the thrombotic sites {Berny, 2010 #259; Nagy, 2017 #18}. Using flow chamber devices, in only a limited number of papers the combination of platelet activation and coagulation has been investigated (Figure 2b). To assess these processes simultaneously, microfluidic experiments under coagulating conditions are performed on coatings with collagens often in the presence of tissue factor (TF). Microspot co-coating of collagen and TF triggers the factor VII-dependent activation of the extrinsic coagulation pathway [44,45], whereas coating with only collagens causes the factor XII-dependent activation of the intrinsic pathway [9]. In either case, the result is initial adhesion of single platelets that trap other platelets to form thrombi (aggregates), which provide the surface for fibrin fibre formation in a TF-enhanced way (Video S1). The relative amounts of platelet deposition and fibrin formation (due to coagulation) can be influenced, for instance by lowering the collagen coating [13]. In addition, by titrating the coated TF amount, the extent of coagulation triggering can be fine-tuned [46,47].

In the setting of collagen/TF-induced thrombus formation, the generation of procoagulant platelets appears to be crucial for the later growth of a fibrin clot [13]. At high haematocrit levels, the role of red blood cells again is to direct the flowed platelets towards the chamber surface. Accordingly, in reconstitution experiments where diluted blood is supplemented with platelets or red blood cells, both platelet deposition and fibrin formation are enhanced by either intervention [13]. To suppress undesired blood coagulation in the fluid phase of the microfluidic system, several methods can be applied. Commonly used anticoagulants are trisodium citrate and corn trypsin inhibitor (CTI). When using citrate-anticoagulated blood, recalcification is needed to allow coagulation activation [21]. With CTI-treated blood, which suppresses the intrinsic coagulation pathway, recalcification is not required, but the blood samples cannot be stored very long [46]. For a detailed list of published studies performed with blood taken on specific anticoagulants, we refer to an earlier review [21].

Different shear rates must be applied to mimic the platelet-dependent coagulation process at venous, arterial or stenotic (pathologically high) shear conditions, as detailed elsewhere [21]. Varying the blood shear and flow rates markedly affects the formation of platelet-fibrin clots. A higher wall-shear rate evokes stronger VWF-dependent platelet adhesion, while the accompanying higher flow rate causes more dilution of the locally generated thrombin [21]. In the circulation, vascular obstruction by a thrombus can be bypassed by collateral blood vessels. An adaptation to mimic such bypass effects is application of a multichannel pressure drop device [44]. Herein, the flowed blood can escape from occluding channels by redirection to still open channels. In single-channel flow devices that are close to obstruction due to the formation of a fibrin-clotting thrombus, very high shear rates can be obtained, resembling those observed at stenotic arterial sites [4,48]. Another type of flow device where defects in thrombus growth and fibrin formation can be detected is the Total Thrombus-formation Analysis System (T-TAS). Here, the in-system’s pressure is measured as a proxy for thrombus growth. For coagulating purposes, it contains a coating with collagen and TF, while citrated/recalcified or CTI-treated blood can be perfused [47].

**Figure 3:**
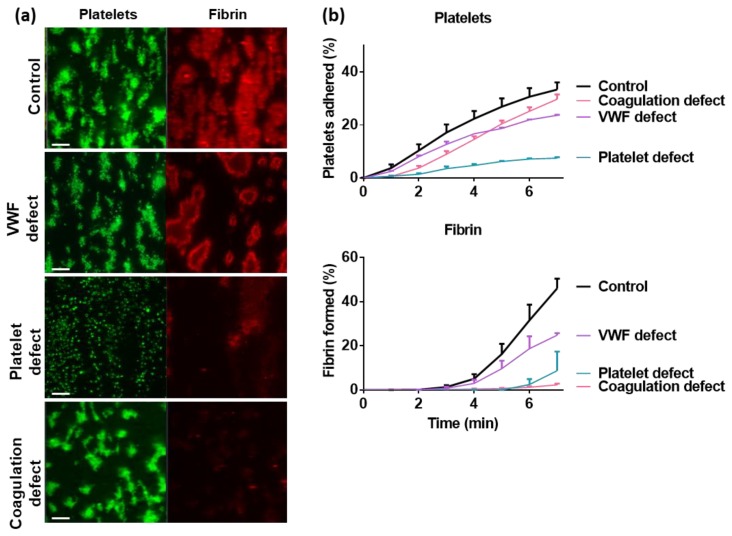
Impaired platelet activation and fibrin formation in blood from patients with bleeding disorders depicted with the Maastricht flow chamber. Citrated blood samples from control subjects or patients with a defect in either VWF, platelet activation or a coagulation factor was recalcified and flowed over collagen at a wall shear rate of 1000 s^−1^. Multicolour fluorescence images were captured every minute to detect platelet deposition (green) and fibrin formation (red). (**a**) Representative images of adhered platelets (green) and fibrin (red) after 10 min of blood perfusion. (**b**) Real-time kinetics of platelet deposition (top) and fibrin formation (bottom) during blood flow. Means ± SEM, n = 3. Figure modified from Refs. [13,53].

Microfluidic assays under coagulating conditions can be used to study abnormalities in the blood from patients with a genetic or acquired platelet or (anti)coagulation disorder. Common procedures for such investigations are described in Box A3. Deficiencies in a range of coagulation factors and several platelet disorders can be picked up in this way [4,21]. In Figure 3 it is illustrated that a given platelet defect and to a lesser degree a VWF defect results in diminished thrombus and fibrin formation in comparison to the control blood. On the other hand, a severe coagulation defect can lead to abrogation of the fibrin formation.

## 5. Whole Blood Microfluidics to Study Fibrinolysis

Fibrin clots produced by the coagulation cascade have a limited life-time, both in vivo and *in vitro*, due to the activity of fibrinolysis. The fibrinolytic pathway consists of a number of protein factors, co-factors, receptors and inhibitors [7]. Platelets with fibrin contribute to this process, as well as the physical properties of a clot and the overall biochemical environment [49,50]. A key fibrinolytic factor is plasminogen, which is cleaved into plasmin by tissue or urokinase plasminogen activator (tPA or uPA). The latter protease actively degrades fibrin fibres. Microfluidics studies have indicated that, under flow conditions, the lysis of a fibrin clot is greatly promoted by the local presence of tPA or uPA, which preferentially is included during the growth of platelet-fibrin thrombus. In addition, fibrinolysis is facilitated by the inhibition of thrombin or by higher flow rates [51]. Microfluidic devices that allow to determine the kinetics of this process may have the potential to better assess the fibrinolytic potential of a blood sample. Given the fact that the fibrinolytic pathway is enforced by blood flow, such microfluidic tests will provide an advantage over static point-of-care fibrinolysis tests, using rotational thromboelastometry or thromboelastography [52].

## 6. Microfluidics to Study Whole Blood Interactions with Endothelial Cells

As an extension of microfluidic chambers coated with collagen, more comprehensive models are being developed of ‘endothelialised’ devices. These allow to study in vitro how endothelial cells affect blood-borne processes in a flow environment (Figure 2c). A major advantage of such devices is that multiple experimental variables can be modified in a controlled way, such as channel geometry, shear rate, coagulation potential, and the endothelial activation or inflammatory state [19]. The possibility of change the design makes such vessel-on-a-chip devices as attractive model systems to study the complex interactions between blood components and the vessel wall. On the other hand, the current variability in system design retards efforts to standardisation.

When establishing an in vitro endothelial model system, the choice of the cell line is important. Most frequently used in microfluidic studies are human umbilical vein endothelial cells (HUVEC) [54,55,56,57,58,59,60,61,62]. As essentially primary cells, when applied at low passage numbers, HUVEC-covered surfaces have served to study several aspects of vascular function, with as advantages a high experimental reproducibility and a lack of contamination of non-endothelial cells.

When employed at resting condition, a monolayer of HUVEC incorporated into flow chambers suppress the adhesion and activation of flowed platelets (Figure 2c). On the other hand, activation of the HUVEC in several ways allows assessment of shear-dependent, VWF-mediated platelet adhesion. Various laboratories have shown that ultra-large VWF multimers secreted by HUVEC can effectively trap platelets at high-shear conditions. This VWF release from Weibel-Pallade bodies can be stimulated by a range of agents stimulating the endothelial cells (Table 1). Additionally, using HUVEC, it was demonstrated that flow-shear gradients in a microfluidic channel induce considerable heterogeneity in VWF release with as a consequence locally higher platelet accumulation at post-stenotic sites [48]. Targeted activation of HUVEC with tumour necrosis factor-α also results in increased platelet adhesion as well as in moderate generation of thrombin and fibrin on the cell surface [63,64]. In certain circumstances, it is desirably to use endothelial cell lines other than HUVEC. For instance, cultured lymphatic endothelial cells, known to express high levels of podoplanin (ligand of CLEC-2 receptor), have been used to study CLEC-2 mediated platelet adhesion under flow. In this case, quiescent HUVEC were used as negative control cells [59].

Employing a mechanical injury model, consisting of an endothelialised microfluidic system coupled to a micro-engineered pneumatic valve, it was found that HUVEC and HAEC (human aortic endothelial cells) responded differently to certain variations in shear rate [61]. Upon whole-blood perfusion, fibrin formation was more prominent at lower shear rates in the presence of HUVEC than of HAEC. Certain papers described the use of a confluent monolayer of endothelial cells that were fixed prior to blood perfusion [59,64]. Although these fixed cells had retained the functional expression of adhesive receptors, it should be remarked that a ‘real’ endothelium is not acting as a passive barrier, but rather as a monolayer of highly active cells.

Human blood outgrowth endothelial cells (BOEC) derived from individual patients with endothelial-related disorders provide a promising tool for the understanding and treatment of cardiovascular diseases. The BOEC are cultured from blood-borne endothelial progenitor cells, and can be used in microfluidic devices. For instance, BOEC derived from patients with type 3 von Willebrand disease (a congenital deficiency in endothelial-derived VWF expression) appeared to be dysfunctional in the release of large VWF multimers that capture platelets under flow conditions [68]. An approach to use HUVEC in a clinically relevant manner is by subjecting these to blood or plasma samples with a reduction in ADAMTS-13 (which cleaves and inactivates the ultra-large VWF multimers), obtained from patients with coronary artery disease [60]. A particular observation was that, under flow, the recruitment of monocytes to adhered platelets is enforced by an endothelial release of large VWF multimers, a process that is exacerbated by a moderate ADAMT-13 deficiency. Additional approaches to use a partly dysfunctional or inflamed endothelium are reviewed by others [19].

In recent years, even more complex approaches being referred to as human organoid-on-a-chip devices are emerging as tools to predict pathophysiological aberrations at the organ level. The model organoid devices commonly contain organ-specific three-dimensional structures encapsulated in small chambers. One example is a device that mimics the alveolar-capillary interface involved in pulmonary thrombosis [69]. This multi-layered microfluidic device consists of two parallel microchannels lined with either human lung alveolar epithelial cells or HUVEC, the two of which are separated by a porous elastic membrane coated with extracellular matrix components. The authors could monitor shear-dependent platelet deposition and thrombus morphologies on endothelial cells with inflamed characteristics. It was furthermore observed that lipopolysaccharide endotoxin-induced activation of the epithelial cells triggered prothrombotic reactions in the adjacent endothelial cells, thus demonstrating novel tissue-tissue interactions in the context of intravascular pulmonary thrombosis. Such organoids-on-a-chip may also pave the way for a next level preclinical testing of potential novel therapeutics.

Taken together, it appears that vascularised microfluidic chips provide a way to address multiple research questions that cannot be assessed by the traditional, static tests. However, there is now a need for standardised approaches —e.g., regarding cell tissue source, passage number, growth and treatment— for future improved assessment of clinical abnormalities in mechanisms that regulate platelet-endothelium and coagulation-endothelium interactions in thrombotic and bleeding diseases.

## 7. Towards Standardisation and Clinical Use

Over the years, several improvements have been made on microfluidic devices to better tailor the whole-blood flow assays for specific research questions. Although this has advanced our insight into the mechanisms of blood-borne processes, the research has led to a wide diversification of flow chambers, channels and coatings. For a consistent, wide-spread use in the clinical laboratory, efforts are needed to reduce this diversification and thereby improve inter-lab reproducibility and quality of the measurement outcomes. A first onset to come to standard settings for pre-clinical investigations was made in 2011 by the Scientific Standardisation Committee of the International Society on Thrombosis and Haemostasis. Identified were as main sources of variability: the coating surfaces, the type of perfusion chamber, the output measurements (including data analysis), the type of blood anticoagulant, and the blood storage time between collection and experiment [42]. Whereas progress has been made, for example in the development of semi-automated image processing scripts, it is still challenging to come to a consensus in for instance (disposable) flow-channel construction and in blood handling [29]. In general we foresee that publication of technical and handling protocols via open access web-sites will be beneficial.

Research has indeed shown that assay standardisation is an essential step for future use in the clinic [70]. To prelude on this, in a recent study, we used a highly standardised microspot-based multiparameter assay employing flowed blood, which allowed to reveal parameters of subtle inter-individual variability of platelet function in thrombus formation in a cohort of 94 genetically defined subjects [32]. The standardisation aspects included: usage of a high precision mould for microspot coating; a uniform flow channel; strict control of blood drawing, anticoagulants and storage of samples; defined time protocols for blood flow, rinsing, staining and capturing of microscopic images; and a semi-automated, blind data analysis. This effort has resulted in a large and consistent dataset of meanwhile several hundreds of subjects, which defines normal variation in all outcome values, and can act as a base for subsequent studies with patient blood samples. In general, it appears that minimisation of the technical, pre-analytical (blood quality) and test variables allows better discrimination between normal (healthy) and aberrant (disease) test outcomes.

Given the above, the use of microfluidic chips for clinical diagnostics is still in its infancy with only a few patient groups being examined so far [4,71,72]. Nevertheless, several characteristics have indicated that the microfluidic technique is advantageous over conventional tests in the diagnostic laboratory, such as light transmission aggregometry [73,74], flow cytometry [75] and platelet function analysers [76]. Whole-blood flow assays uniquely integrate the additive contributions of haemodynamic forces, platelet activation and coagulation to the process of thrombus formation. Furthermore, they require only small amounts of unprocessed blood, and generate high throughput outcome values. All of these characteristics render microfluidic assays suitable for detecting small changes in the ‘haemostatic and thrombotic’ activities of a blood sample and, hence, for tracking subtle differences in platelet or coagulation responses to appropriate stimuli [4]. These aspects are relevant *per excellence* for a tailor-made monitoring of patient responses to (combinations) of prescribed antithrombotic drugs.

## Figures and Tables

**Table 1:** Microfluidic models used to assess platelet deposition on ultra-large multimers released by activated or injured HUVEC.

Endothelial Injury	Surface	Shear Rate	Output Measurement	Ref.
Histamine, sCD40L, bradykinin; activated platelets	gelatin	2.5–10 dyne/cm^2^	platelets on large VWF multimers	[60,65]
TNF-α	not specified	2.5 dyne/cm^2^	platelets on-VWF strings	[56]
Histamine	not specified	1–2.5 dyne/cm^2^	VWF strings	[57]
Phorbol myristate acetate	rat tail collagen	10–30 dyne/cm^2^	VWF strings, platelet adhesion	[61,65]
Stenosis	rat tail collagen	1000 s^−1^	platelet aggregation	[54]
Mechanical injury	collagen type I	500 or 2500 s^−1^	platelets adhered to VWF	[61]
Tumor supernatant	gelatin	venous	ULVWF multimer secretion, VWF-platelet strings length	[66]
Histamine	gelatin	2.5–50 dyne/cm^2^	platelets on VWF-strings	[67]

## Supplementary Materials

The following are available online at https://www.mdpi.com/2072-666X/10/11/787/s1, Video S1: Whole-blood formation of platelet-fibrin thrombi on a collagen/TF microspot at wall-shear rate of 1000 s^−1^. Red colour represents localised deposition of Alexa Fluor-647 labelled fibrin. Total time is 10 min.

## Appendix A

Box A1 Experimental Outline for Whole Blood Flow by Pushing High-Precision Fluid Pumps. Provided that the flow pattern is parabolic, the wall-shear rate *γ* of flowing blood in a parallel-plate chamber is described by Equation (A1):

*γ* = 6 *Q*/*h^2^w* (A1)

Flow simulations indicate that a parabolic flow pattern is obtained when the width of parallel-plate chambers exceeds the height by far [40]. In this equation, *Q* is the volumetric flow rate (mm^3^/s), *h* is the chamber height (mm), and *w* the chamber width (mm). The wall shear rate *γ* accordingly is given in inverse seconds (s^−1^). Calculation of the *shear stress* (*τ*) requires knowledge of the viscosity (*η*), according to Equation (A2):

*τ* = *η* × *γ* (A2)

Note that both equations assume that the blood acts as a Newtonian fluid. In reality, the blood viscosity varies through the flow chamber, because under flow red blood cells will concentrate in the centre of the blood stream, resulting in platelet accumulation close to the chamber walls. By definition, the dimensions of a parallel-plate flow chamber determine the volume of blood, required for experiments at a defined wall-shear rate. In common use, fluid consumption for the Maastricht chamber of 50 μm height and 3.0 mm width is as follows. For medium to high (arterial) shear rates: 300 μL blood for a 4 min perfusion at 1000 s^−1^; and 480 μL blood for a 4 min perfusion at 1600 s^−1^ [29]. Concerning low (venous) shear rates: 113 μL blood is required for a 10 min perfusion at 150 s^−1^. During single-pass blood flow through the chamber, the build-up of thrombi will gradually obstruct the chamber lumen, and hence locally change the wall shear rate and flow pattern. For control of the flow rate in a microfluidic parallel-plate chamber, a high-precision perfusion pump needs to be used. Advantages of pushing the blood (placed in a plastic syringe) through the chamber is that a better control is obtained of leakage at the coverslip, and that the formation of air bubbles is minimal. On the other hand, an advantage of syringe pulling is that a blood sample can be drawn from semi-closed tubes and finally collected into a reservoir, thus limiting blood contact with the environment. Common use is to perfuse a blood sample at constant flow rate, but in specific cases pulsatile changes in flow rate are preferred, reflecting the regular flow changes triggered by a pumping heart.

Box A2 Experimental Outline for Whole Blood Thrombus Formation without Coagulation. *1. Anticoagulation control.* Adequate control of the blood coagulation is needed for reproducible flow measurements aimed to study platelet activity. Since platelet adhesion and aggregation are dependent on millimolar levels of free Mg^2+^ and Ca^2+^, samples of citrate-anticoagulated blood preferentially are supplemented with a MgCl_2_/CaCl_2_ mixture, after the addition of a thrombin inhibitor (PPACK or hirudin). For the rapidly coagulating mouse blood, heparin preferably is added as well. Several precautions can be made to suppress a residual coagulation. these include a pre-incubation of drawn blood samples at 37 °C in order to inactivate traces of autocrine ADP and thrombin, both causing sensitisation and activation of the platelets. An indication of insufficient anticoagulation in recalcified blood samples is a lowered platelet count. During blood perfusion through the microfluidic chamber, residual coagulation is apparent from the passing of large platelet-fibrin clots. Obviously, platelet adhesion diminishes in partially clotted blood samples. Formation of air bubbles in the chamber must be prevented, since these cause platelet necrosis and trigger the clotting process. *2. Microspotted platelet-adhesive coatings.* By applying a micospot coating of 1 μm in diameter, e.g., of vascular components like collagen type-I or laminin, several platelet-adhesive surfaces can be investigated at the same time during one flow run [21,30]. The combined use of several microspots allows high throughput analysis of platelet functions. *3. Microscopic imaging and analysis.* A standard way to evaluate platelet deposition, activation and aggregate formation during whole blood flow is by using a multicolour fluorescence microscope with state-of-the-art optics and equipped with a sensitive camera [29,77]. Software using defined image analysis scripts is available for valuable, multiparameter output [32].

Box A3 Experimental Outline for Whole Blood Thrombus Formation with Coagulation. For use with the Maastricht chamber, cleaned glass coverslips (24 mm × 60 mm) are coated with microspots of 1 μL collagen type-I (50 µg/mL) and, if desired, with 1 μL tissue factor (TF, 500 pM) [29,77]. Blood is collected by venepuncture into 3.2% trisodium citrate. A sample can be mixed with anticoagulant to prevent the generation of traces of thrombin. Alternatively, blood samples are collected into saline containing corn trypsin inhibitor (CTI), which prevents the contact activation pathway. Desired antagonists and fluorescent probes can be added to a sample of 0.5 mL prior to the flow run [29]. Several procedures can be used to trigger coagulation in a controlled way. Blood collected into CTI should be directly used after drawing. Alternatively, citrated whole blood can be co-perfused with a CaCl_2_/MgCl_2_ mixture. Another approach is the premixing of a blood sample with CaCl_2_/MgCl_2_ mixture directly before the flow experiment; this may require the inhibition of contact activation, e.g., with CTI. Using multicolour fluorescence microscopy, platelet deposition and fibrin formation can be quantified by microscopic imaging at the same time (see Video S1).

## Appendix B

**Table A1:** Representative microfluidic devices and protocols used to study platelet deposition under flow.

Device	Geometry	Shear rate	Anticoagulant	Sample	Thrombogenic Surface	Protocol	Coag.	Ref.
Maastricht flow chamber	d = 50 μm, w = 3 mm, l = 30 mm	150 s^−1^, 1000 s^−1^, 1600 s^−1^	Citrate, PPACK, fragmin	Whole blood	52 surfaces	4–6 min perfusion, image acquisition, staining, acquisition	No	[30]
Maastricht flow chamber	d = 50 μm, w = 3 mm, l = 30 mm	1000 s^−1^	Citrate	Whole blood	6 surfaces	3.5 min perfusion, staining, image acquisition, rinsing, acquisition	No	[32]
Parallel channels with stenoses	d = 0.18 mm, w = 0.2–1 mm, l = 70 mm	Calculated	Citrate, PPACK	Whole blood	VWF, fibrinogen	5 min perfusion, rinsing, fixation, image acquisition	No	[78]
PDMS, 8 channels flow device	n.i.	100 s^−1^	CTI	Whole blood	Collagen, TF	15 min perfusion, image acquisition every min	Yes	[79]
PDMS, 8 channels flow device	d = 60 μm, w = 250 μm	200 s^−1^	PPACK	Whole blood	Collagen	5 min perfusion, image acquisition	No	[80]
Parallel plate flow chamber	d = 120 µm, w = 450 µm, l = 2 cm	300 s^−1^	Heparin	Whole blood	Collagen	3 min perfusion, rinsing, image acquisition	No	[81]
PDMS ladder network	Main channels: w = 100 µm, d = 100 µm, Bypass channels: w = 50	Variable	Citrate	Whole blood	Collagen	30 min perfusion, rinsing, image acquisition	No	[82]
Maastricht flow chamber	d = 50 μm, w = 3 mm, l = 30 mm	150 s^−1^, 500 s^−1^, 1000 s^−1^	Citrate	Whole blood	Collagen/TF, plaque material	10 min recalcified, image acquisition every 2 min	Yes	[13,83,84]
PDMS vs. Ibidi sticky-slide I 0.1	PDMS: d = 60 μm, w = 250 μm vs. Ibidi	1400 s^−1^	Hirudin	n.i.	Collagen	Perfusion, image acquisition	No	[85]
Ibidi μ-slide-I 0.1 Luer	d = 100 μm, w = 5 mm, l = 60 mm	1500 s^−1^	n.i.	PRP	Collagen	5 min perfusion, rinsing, image acquisition	No	[86]
PDMS, 8 channels flow device	d = 60 μm, w = 250 μm	100 s^−1^, 1000 s^−1^	CTI	Whole blood	Collagen ± TF, VWF	Perfusion, image acquisition	Yes	[87]
Laser cut PSA	d = 50 μm, w = 2 mm, l = 75 mm	1500 s^−1^	Citrate	Whole blood	VWF	Perfusion, image acquisition	No	[88]
Well plate device	n.i.	250 s^−1^, 5000 s^−1^	Citrate	Whole blood	Collagen, VWF	2–5 min perfusion, image acquisition	No	[89]

*Abbreviations:* Coag., coagulation; d, depth, w, width, l, length; PPACK, _D_-phenylalanyl-_L_-prolyl-_L_-arginine chloromethyl ketone; PRP, platelet rich plasma; VWF, Von Willebrand factor; PDMS, polydimethylsiloxane; CTI, corn trypsin inhibitor; TF, tissue factor; PSA, pressure-sensitive adhesive; n.i., not indicated.
